# The Case of the Fickle Fingers: How the PRDM9 Zinc Finger Protein Specifies Meiotic Recombination Hotspots in Humans

**DOI:** 10.1371/journal.pbio.1001211

**Published:** 2011-12-06

**Authors:** Laure Ségurel, Ellen Miranda Leffler, Molly Przeworski

**Affiliations:** 1 Department of Human Genetics, University of Chicago, Chicago, Illinois, United States of America; 2 Howard Hughes Medical Institute, University of Chicago, Chicago, Illinois, United States of America; 3 Department of Ecology and Evolution, University of Chicago, Chicago, Illinois, United States of America

## Abstract

During mammalian meiosis, double-strand breaks are deliberately made throughout the genome and then repaired, leading to the exchange of genetic material between copies of chromosomes. How the locations of breaks are specified was largely unknown until a fortuitous confluence of statistical genetics and molecular biology uncovered the role of PRDM9, a DNA binding protein. Many properties of this protein remain mysterious, however, including how it binds to DNA, how it contributes to male infertility—both in humans, and in hybrid mice—and why, in spite of its fundamental function in meiosis, its binding domain varies extensively among humans and across mammals. We present a brief summary of what has recently been learned about PRDM9 in different fields, focusing on the puzzles yet to be resolved.

## Abstract 2

Recent discoveries have revealed the central role of PRDM9 in mammalian recombination. The precise function of this protein, however, remains poorly understood, as do the causes for its rapid evolution and its role in reproductive isolation.

## Introduction

Homologous recombination refers to the process by which DNA is broken and exchanged between copies of chromosomes. It is essential to the proper alignment and segregation of chromosomes during meiosis, with double-strand breaks serving to initiate the homology search and crossovers (one of the possible resolutions of recombination) tethering homologs together in order to ensure proper disjunction [1]. In humans, as in many mammals, recombination events tend to concentrate in specific segments of the genome (typically <2 kb), referred to as “hotspots”, that are orders of magnitude more likely to experience a break than surrounding regions. We have learned about the characteristics of human hotspots from studying large numbers of pedigrees and from sperm-typing experiments, as well as by using patterns of genetic variation data to infer “historical hotspots”, which reflect population recombination rates averaged over males and females and over ancestral generations.

How hotspot locations and intensities are specified remained obscure until recently, when an epigenetic modification (the tri-methylation of histone H3 on lysine 4, H3K4me3) was shown to be an important mark for the initiation of recombination in yeast and mice [2],[3],[4], and a 13-mer sequence motif (“CCnCCnTnnCCnC”) was found enriched in human historical hotspots as compared to coldspots [5],[6] and shown to modulate crossover activity (e.g., [7]). A series of studies also revealed that, in spite of the essential role of recombination in meiosis, tremendous variation exists in the placement and intensity of crossovers among humans [8],[9], among mice strains [10], and between humans and primates [11],[12],[13],[14]. Mapping the source of this variation led to a breakthrough in our understanding of how hotspots are specified, with the identification of the role of PRDM9.

**Figure 1:**
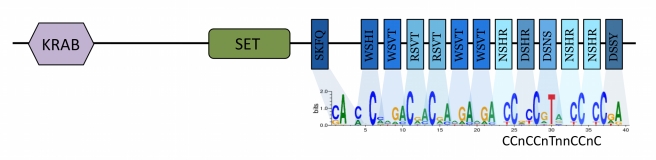
The three domains of PRDM9, along with the binding prediction for the zinc finger array. PRDM9 contains a KRAB domain, which is thought to be involved in transcriptional repression, as well as a SET domain that tri-methylates H3K4, an epigenetic mark associated with the initiation of meiotic recombination in yeast and mice [2],[3],[4]. The zinc fingers are color-coded according to the identity of the residues in contact with DNA. The DNA sequence bound by the zinc finger array of the A variant of PRDM9 was predicted using http://zf.princeton.edu/ (under the polynomial support vector machine model) and aligned with the 13-bp motif found to be enriched in historical hotspots [6].

In 2009, two groups independently associated a region containing *Prdm9* to a difference in recombination activity between mouse strains [15],[16]. This gene was a great candidate [2]: it is expressed only in ovaries and testis [17]; it contains a SET domain that tri-methylates H3K4 and a zinc finger domain able to bind DNA (Figure 1); and *Prdm9*-null mice show arrest of gametes in meiotic prophase I and impaired double-strand break repair [17]. Moreover, the second half of the human PRDM9 zinc finger array is computationally predicted to bind the sequence motifs found enriched in hotspots: specifically, the PRDM9 A variant (86% frequency in Europeans, 50% in African-Americans [18]) was predicted [19] and shown in vitro [20] to bind to the 13-bp motif (see Figure 1), whereas the human C variant (13% frequency in African-Americans, 1% in Europeans [18]) was predicted to recognize the 17-bp motif “CCCCaGTGAGCGTtgCc” enriched in hotspots that tend to be used in African populations but rarely in Europeans [21]. Similarly in mice, the binding prediction for PRDM9 matches a consensus motif overrepresented in hotspots [4] and direct binding has been confirmed in vitro [22]. Experimental and population genetic studies further revealed variation in PRDM9 zinc fingers to have a major impact on the location and intensity of crossovers in humans [18],[21],[23],[24]. Indeed, differences among individuals at PRDM9 explain ∼80% of heritable variation in “hotspot usage”, the fraction of crossovers placed in hotspots genome-wide [20],[21],[25]. Consistent with these findings, in transgenic mice, the introduction of changes to PRDM9 zinc fingers leads to differences in hotspot activity, H3K4me3 levels, and the genome-wide distribution of crossovers [22]. The past couple of years have thus witnessed a remarkable convergence of evidence from different disciplines, suggesting that the locations of breaks are in part specified by DNA motifs to which PRDM9 zinc fingers bind, eventually recruiting the recombination machinery.

In spite of this rapid progress, however, a number of pieces do not fit into the puzzle, notably the tenuous relationship observed in sperm-typing experiments between PRDM9 variants, their predicted motifs, and the resulting recombination activity [18],[22],[24]. We still have little understanding of the role of PRDM9 in double-strand break formation and repair, or of the mechanism through which it helps to initiate recombination. Also mysterious is the observation that PRDM9 zinc fingers evolve exceptionally rapidly among primates and rodents [19],[26]. Finally, *PRDM9* emerged in a completely distinct context: as the first (and to date only) locus shown to underlie hybrid sterility in mammals [27]. Here, we focus on these incongruous pieces, discussing what remains to be understood and suggesting possible resolutions.

## Does PRDM9 Specify All Human Recombination Hotspots?

**Figure 2:**
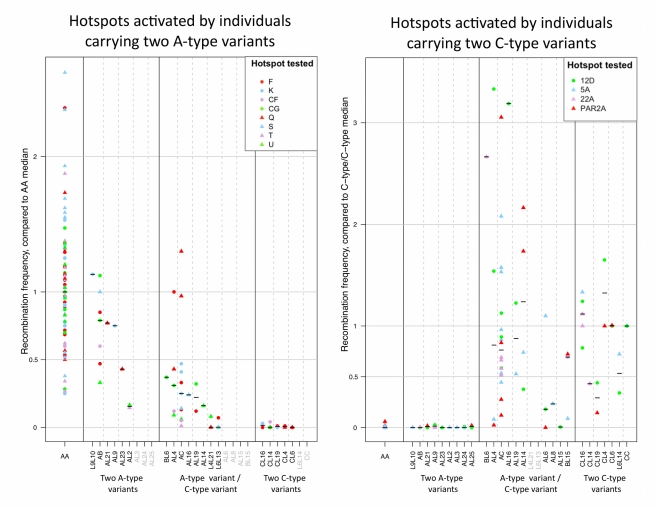
The effect of PRDM9 zinc finger variants on hotspot activity. Each column presents males with the same genotype, grouped according to whether they carry two A-type variants (defined as variants predicted to bind the same 13-bp motif as A), two C-type variants (defined as variants predicted to bind the same 17-bp motif as C), or one A-type and one C-type variant. Within a column, each symbol denotes the recombination activity of a given hotspot for a given individual, with circles indicating hotspots that contain a perfect match to the 13-bp motif (for the left panel) or the 17-bp motif (for the right panel) within 1 kb of their center, and triangles indicating hotspots with no perfect matches. The median recombination frequency is shown as a black bar. As can be seen, there is no clear difference between the activity of hotspots with and without a perfect match to the motif. The recombination frequency is reported relative to the median of AA individuals (left panel) or that of C-type/C-type individuals (right panel). The data were obtained by sperm-typing from [18] (left panel) and [24] (right panel). The E and PAR2 hotspots from [18] were excluded from the analysis because they contain polymorphisms disrupting the central 13-bp motif [42], possibly confounding the effect of variation in PRDM9. The 12B hotspot from [24] was excluded because it was not active in typed C-type/C-type individuals.

The 13-bp motif recognized by the main A variant is neither necessary nor sufficient to drive hotspot activity in humans: it occurs approximately 290,000 times in the genome when fewer than 50,000 hotspots have been inferred. Originally, it was estimated to play a causal role in ∼40% of historical hotspots [6]. Yet individuals heterozygous for the main A variant and the minor I variant (which has a different motif binding prediction than A, as confirmed in vitro) show a ∼70% decrease in historical hotspot usage as compared to AA individuals [20]. This is oddly high: all else being equal, even if the I variant were dominant and led to complete abrogation of binding to the 13-bp motif, the historical hotspot usage should decrease by only 40% [20]. Even more puzzling, two sperm-typing studies showed that the activity of a sample of 17 recombination hotspots are all influenced by the *PRDM9* genotype, *even when the hotspots do not contain an exact match to the 13-bp or the 17-bp motif* ([18],[24]; see Figure 2). Finally, in seven individuals who likely carry two C-type variants (defined as variants predicted to bind the same 17-bp motif as does the C variant), there is no evidence of activity at hotspots defined from linkage disequilibrium patterns or pedigree analyses in Europeans, in which C-types are rare [21]. Together, these observations strongly suggest that PRDM9 influences more hotspots than previously thought, and possibly all of them.

How does PRDM9 influence human hotspots without clear matches to their predicted motif? While the answer could be as simple as binding predictions for PRDM9 being unreliable, it seems unlikely given that they helped lead to the discovery of the role of this gene in human recombination, and were verified in vitro for two variants (A and I) [19],[20]. An alternative is that PRDM9 can bind the degenerate versions of motifs that are ubiquitous in the genome. However, earlier sperm-typing studies showed that single point mutations in the 13-bp motif can completely knock down hotspot activity [7],[28],[29], so this argument leads to the seemingly paradoxical conclusion that PRDM9 is both highly specific and permissive at the same time. Also unclear is whether PRDM9 always influences hotspot activity through direct binding, indirectly, or both [18],[30].

## Incongruities between PRDM9 Variants and Hotspot Activity

PRDM9 zinc fingers are highly diverse among humans, with over 20 variants already described [15],[18],[20],[23], including C-type variants, as well as A-type variants (defined as predicted to recognize the same 13-bp motif as does A). Surprisingly, a sperm-typing study at ten hotspots activated by AA individuals reported that, while on average males carrying one copy of A have 41%+/−16% of the median recombination rate of AA individuals, males carrying one copy of most other A-type variants do not activate any of these hotspots [18]. This observation raises the possibility of salient functional differences between A and other A-type variants. An alternative explanation might be that not all A-type variants are co-dominant in their effects on crossover activity, and some A-type variants are coupled with dominant C-type variants that partially mask their effects.

In order to better understand the dominance relationships, we reanalyzed hotspot activity from previous sperm-typing studies, focusing on A-type and C-type variants (see Table S1, [18],[24]). As shown in Figure 2, A-type/A-type males activate all ten hotspots active in A/A males, but none of the four hotspots active in C-type/C-type males (from [24]); conversely, C-type/C-type males do not activate any of the ten hotspots active in A/A males. Interestingly, the activity of A-type/C-type males is on average not discernibly lower than that of C-type/C-type males for the four hotspots active in C-type homozygous individuals, but is clearly reduced for the ten hotspots active in A/A individuals. This observation suggests that, as a class, C-type variants partially dominate A-type variants in their effects on crossover activity, either directly (e.g., by outcompeting them for binding) or indirectly (e.g., in creating more breaks in the genome). Moreover, the dominance effects appear to depend on the specific combination of variants.

Even so, the large variation in activity seen among A-type and C-type variants for the same set of hotspots remains a puzzle [18],[24]. Perhaps additional variation in the zinc fingers or elsewhere in the protein influences hotspot activity: residues not predicted to be in contact with DNA could affect the stability of binding [18],[20],[31], or the zinc fingers could be involved in binding co-factors required for the function of the protein —whether protein or RNA—as documented for other C2H2 zinc fingers [31]. Alternatively, as in the case of the zinc finger CTCF, the DNA binding motif may be even longer than 13 bp, consistent with the extended motif found to be enriched in historical hotspots [6].

Beyond the zinc fingers, other factors likely influence the location of double-strand breaks, including chromatin accessibility, competition among motifs in close proximity, co-factors acting in a multi-protein complex, or additional epigenetic marks [8],[32],[33]. In this respect, we note that little is understood about variation in the “penetrance” of the motif on different genetic backgrounds; for example, why the 13-bp motif is nearly 50 times more likely to be associated with a hotspot when it lies in the context of a THE1B repeat than when it is on a non-repeat background [6]. Additional uncharacterized variation in *cis* (e.g., polymorphisms in a motif) can also affect binding affinity of PRDM9 and could contribute to the variability seen among individuals (e.g., [22],[24]).

## Insights from the Role of PRDM9 in Sterility

Crosses among species can reveal deleterious interactions among alleles (termed “Muller-Dobzhansky incompatibilities”) that had never segregated together in the same population (e.g., [34]). F1 offspring of certain crosses of *Mus mus domesticus*×*Mus mus musculus* show meiotic arrest in prophase due to a Muller-Dobzhansky incompatibility involving *Prdm9* together with the X chromosome [27]. This incompatibility appears to be due to the different alleles segregating in mice subspecies: the Hst1*^s^* (for sterility) and Hst1*^f^* (for fertility) variants of the zinc fingers of PRDM9 from *M. mus domesticus* and the Hst*^ws^* and Hst*^wf^* alleles (putatively also at *Prdm9*) in *M. mus musculus* [35]. It manifests itself only in males carrying an X chromosome from *M. mus musculus* together with Hst1*^s^* and Hst*^ws^* at *Prdm9*; all other combinations of *Prdm9* alleles are fertile, as are female F1 ([27]; J. Forejt, personal communication). Moreover, male sterility can be rescued by introducing additional copies of the Hst1*^f^* allele [27]. That only Hst1*^s^*/Hst*^ws^* leads to sterility points to dosage-sensitivity as well as to deleterious interactions between some variants at PRDM9, as could happen, for example, if PRDM9 forms a homodimer (cf. [36]). Thus, studies of reproductive isolation, although not focused on recombination phenotypes, support the hypothesis of complex interactions between PRDM9 variants.

We note that, within a single subspecies, mice carrying the sterility allele are fertile [27]. Thus, there is no reason to assume that, in the absence of a deleterious interaction with another locus, heterozygosity at *PRDM9* per se compromises fertility within humans (contrary to [37]). Loss-of-function alleles could lead to sterility, however, as seen in mice [17]—in which case the variant should be kept at very low frequency by natural selection. Variants in PRDM9 could also be associated with more subtle effects on fertility. Consistent with this hypothesis, a resequencing study of *PRDM9* in infertile and fertile Japanese men found that the minor alleles of three SNPs in the zinc finger domain (two of which alter residues in contact with DNA) were significantly enriched among fertile men [38]. Given our increased understanding of PRDM9, a larger study of this kind would be opportune.

## Why Does the Zinc Finger Evolve So Rapidly?

The residues of PRDM9 zinc fingers in contact with DNA show an unusually high rate of change in both rodents and primates [19],[26], strongly suggesting repeated bouts of positive selection for novel binding targets. Why might this be? One idea is that the zinc finger changes repeatedly in order to counteract the inherent self-destructive property of hotspots. The argument is as follows: Double-strand break repair uses the intact homolog as a donor of information, with the consequence that, in heterozygous individuals, alleles more likely to experience a break tend to be converted to “colder” alleles. Over evolutionary time, hotter alleles are therefore doomed to extinction, along with their associated hotspots [39],[40],[41]. Consistent with this model, the 13-bp motif has been lost from the human lineage faster than in the chimpanzee lineage, in which it does not seem to be active [19]. The loss of individual hotspots could eventually imperil alignment and segregation, creating a selective pressure to recognize novel target sequences and selecting for new PRDM9 variants [19],[20],[39]. Whether this scheme is realistic remains to be modeled.

Alternatively, the zinc finger could be evolving rapidly unrelated to its role in recombination per se: for example, PRMD9 could have a role in suppressing selfish elements in the genome [19]. Its rapid evolution could also be related to its possible role as a transcriptional regulator (e.g., [27]).

## Towards a Solution

Some of the incongruous observations might be explained if PRDM9 is responsible for the specification of all or almost all hotspots; if PRDM9 variants interact with one another and are dosage sensitive, and if the first half of the zinc fingers also affects binding. What is now required is a diverse set of experiments contributed from many fields, ranging from structural and molecular biology to speciation and evolutionary biology. Further knowledge about the structure of PRDM9, its binding properties and its possible cofactors, as well as its characterization in other species, will then allow us to address questions raised by recent findings, notably: Given the hundreds of thousands of motif instances in the genome to which PRDM9 could bind, how are recombination hotspots specified? How does the zinc finger evolve to find new motifs without deleterious effects on alignment and segregation, and what are the constraints on the state space of possible motifs? Is its rapid change due specifically to its role in recombination or is the change in hotspot activity a pleiotropic consequence of some other function [37]? Is variation in the PRDM9 zinc fingers repeatedly involved in hybrid sterility among species [26]? The story of PRDM9 nicely illustrates the benefits of integrating approaches from many disciplines. Conversely, cracking the curious case of PRDM9 promises to provide important insights into large swaths of biology, from human genetics to speciation.

## Supporting Information

Table S1 The recombination activity of different variants at PRDM9.
(DOC)
