# Supplementary material for: The Case of the Fickle Fingers: How the PRDM9 Zinc Finger Protein Specifies Meiotic Recombination Hotspots in Humans
Source: PLoS Biol. 2011 Dec 6;9(12):e1001211. doi: 10.1371/journal.pbio.1001211 (PMC3232208; doi:10.1371/journal.pbio.1001211)
Supplement: Table S1 — The recombination activity of different variants at PRDM9. (DOC) [file pbio.1001211.s001.doc]

| **Genotype** | **N** | **Relative hotspot**  **activity from [18]^1^** | **Relative hotspot**  **activity from [24]^2^** |
| --- | --- | --- | --- |
| **AA** | 88/40 | 1.00 | 0.00 |
| **AL5** | 2/0 | 1.47 | NA |
| **L9L10** | 1/1 | 1.13 | 0.00 |
| **AE** | 7/0 | 0.81 | NA |
| **AB** | 7/7 | 0.79 | 0.00 |
| **AL21** | 1/2 | 0.77 | 0.01 |
| **AL9** | 1/2 | 0.75 | 0.01 |
| **AL20** | 8/0 | 0.62 | NA |
| **AL23** | 1/2 | 0.43 | 0,00 |
| **BL6** | 1/1 | 0.37 | 2.66 |
| **AL7** | 2/0 | 0.34 | NA |
| **AL11** | 1/0 | 0.32 | NA |
| **AL4** | 5/4 | 0.31 | 0.81 |
| **AC** | 11/18 | 0.25 | 0.76 |
| **AL16** | 1/1 | 0.24 | 3.19 |
| **AL19** | 2/2 | 0.22 | 0.88 |
| **AL2** | 2/1 | 0.16 | 0.00 |
| **AL14** | 1/4 | 0.16 | 1.24 |
| **L11L15** | 1/0 | 0.10 | NA |
| **L4L22** | 1/0 | 0.02 | NA |
| **CL16** | 2/1 | 0.02 | 0.43 |
| **CL19** | 2/2 | 0.01 | 0.29 |
| **CL4** | 2/2 | 0.01 | 1.32 |
| **L17L18** | 1/0 | 0.00 | NA |
| **AL1** | 2/0 | 0.00 | NA |
| **CL6** | 2/2 | 0.00 | 1.00 |
| **EL20** | 2/0 | 0.00 | NA |
| **L6L7** | 2/0 | 0.00 | NA |
| **L4L21** | 3/0 | 0.00 | NA |
| **L6L13** | 4/0 | 0.00 | NA |
| **CL14** | 8/5 | 0.00 | 1.12 |
| **CL12** | 0/1 | NA | 1.59 |
| **CC** | 0/2 | NA | 1.00 |
| **BL15** | 0/3 | NA | 0.69 |
| **L6L14** | 0/2 | NA | 0.53 |
| **AL15** | 0/1 | NA | 0.23 |
| **AL6** | 0/3 | NA | 0.18 |
| **AL22** | 0/1 | NA | 0.04 |
| **AL25** | 0/2 | NA | 0.01 |
| **AD** | 0/5 | NA | 0.00 |
| **AL24** | 0/4 | NA | 0.00 |
| **AL8** | 0/1 | NA | 0.00 |
| **AL3** | 0/1 | NA | 0.00 |

**Supplementary Table 1: The recombination activity of different variants at PRDM9.**

The median recombination frequency of males with similar genotypes is shown for 8 hotspots active in AA males (from [18]) and for 4 hotspots active in males with two C-type variants (from [24]) (see Figure 2 for the subset of hotspots studied). Variants in red correspond to A-type variants and those in blue to C-type variants, while cases in purple denote males carrying one A-type variant together with one C-type variant.

N: number of observations (single value per hotspot per individual). **^1^** Ratio of hotspot activity of males with a given genotype when compared to the median activity of AA males. **^2^** Ratio of hotspot activity of males with a given genotype when compared to the median activity of males carrying two C-type variants.
